# Mitochondrial Haplotypes Associated with Biomarkers for Alzheimer’s Disease

**DOI:** 10.1371/journal.pone.0074158

**Published:** 2013-09-11

**Authors:** Perry G. Ridge, Andre Koop, Taylor J. Maxwell, Matthew H. Bailey, Russell H. Swerdlow, John S. K. Kauwe, Robyn A. Honea, for the Alzheimer’s Disease Neuroimaging Initiative

**Affiliations:** 1 Department of Biology, Brigham Young University, Provo, Utah, United States of America; 2 ARUP Institute for Clinical and Experimental Pathology, Salt Lake City, Utah, United States of America; 3 Kansas University Alzheimer’s Disease Center, Department of Neurology, University of Kansas School of Medicine, Kansas City, Kansas, United States of America; 4 Human Genetics Center, University of Texas School of Public Health, Houston, Texas, United States of America; McGill University Department of Neurology and Neurosurgery, Canada

## Abstract

Various studies have suggested that the mitochondrial genome plays a role in late-onset Alzheimer’s disease, although results are mixed. We used an endophenotype-based approach to further characterize mitochondrial genetic variation and its relationship to risk markers for Alzheimer’s disease. We analyzed longitudinal data from non-demented, mild cognitive impairment, and late-onset Alzheimer’s disease participants in the Alzheimer’s Disease Neuroimaging Initiative with genetic, brain imaging, and behavioral data. We assessed the relationship of structural MRI and cognitive biomarkers with mitochondrial genome variation using TreeScanning, a haplotype-based approach that concentrates statistical power by analyzing evolutionarily meaningful groups (or clades) of haplotypes together for association with a phenotype. Four clades were associated with three different endophenotypes: whole brain volume, percent change in temporal pole thickness, and left hippocampal atrophy over two years. This is the first study of its kind to identify mitochondrial variation associated with brain imaging endophenotypes of Alzheimer’s disease. Our results provide additional evidence that the mitochondrial genome plays a role in risk for Alzheimer’s disease.

## Introduction

While late-onset Alzheimer’s disease (AD) is highly heritable, much of the genetic variance associated with late-onset AD is unknown. Studies of maternal inheritance, mitochondrial genome (mtDNA) mutations, and cytochrome oxidase deficits in AD provide converging evidence for the role of mitochondria in risk for Alzheimer’s disease [1]–[7]. A growing number of studies have reported associations between mitochondrial haplogroups, or specific mitochondrial DNA single nucleotide polymorphisms (SNPs) with risk for AD, however the resulting literature is mixed, either because of differences in the populations studied, sample sizes, or variations in the AD groups studied (reviewed in [8]).

Recently, investigators have developed neuroimaging methods to clarify the role of genes in AD pathology [9]–[12]. The use of endophenotypes, or disease markers, has proved to be sensitive in delineating genetic effects on susceptibility to AD, as the imaging markers provide a more direct measure of the impact of the gene at the level of neuroanatomy. Volumetric magnetic resonance imaging (MRI) has been used to characterize accelerated rates of regional and whole brain atrophy and predict progression to AD before onset [13]–[15]. While a host of studies have used imaging markers to test for relationships between nuclear genes and AD risk [16]–[22], none have used this approach to study mitochondrial genes. The current study sought to clarify the relationship between mtDNA genetic variation and neurodegeneration, a known risk marker for AD.

## Methods

### Subjects

We used publically available data from the Alzheimer’s Disease Neuroimaging Initiative (ADNI) database (adni.loni.ucla.edu), which has undergone rigorous quality control, in the preparation of this article. The ADNI was launched in 2003 by the National Institute on Aging (NIA), the National Institute of Biomedical Imaging and Bioengineering (NIBIB), the Food and Drug Administration (FDA), private pharmaceutical companies and non-profit organizations, as a $60 million, 5- year public-private partnership. The primary goal of ADNI has been to test whether serial magnetic resonance imaging (MRI), positron emission tomography (PET), other biological markers, and clinical and neuropsychological assessment can be combined to measure the progression of mild cognitive impairment (MCI) and early Alzheimer’s disease (AD). Determination of sensitive and specific markers of very early AD progression is intended to aid researchers and clinicians to develop new treatments and monitor their effectiveness, as well as lessen the time and cost of clinical trials.

The Principal Investigator of this initiative is Michael W. Weiner, MD, VA Medical Center and University of California – San Francisco. ADNI is the result of efforts of many co-investigators from a broad range of academic institutions and private corporations, and subjects have been recruited from over 50 sites across the U.S. and Canada. The initial goal of ADNI was to recruit 800 subjects but ADNI has been followed by ADNI-GO and ADNI-2. To date these three protocols have recruited over 1500 adults, ages 55 to 90, to participate in the research, consisting of cognitively normal older individuals, people with early or late MCI, and people with early AD. The follow up duration of each group is specified in the protocols for ADNI-1, ADNI-2 and ADNI-GO. Subjects originally recruited for ADNI-1 and ADNI-GO had the option to be followed in ADNI-2. For up-to-date information, see www.adni-info.org.

Data used in the preparation of this article were obtained from the Alzheimer’s Disease Neuroimaging Initiative (ADNI) database (adni.loni.ucla.edu). Data for the present analysis were downloaded from the ADNI web site in December 2011.

The study reported here involved 821 subjects who had genetic and cognitive data, and MRI scans at least at baseline, and some at 24 months. Of those subjects, 103 subjects were excluded from the FreeSurfer dataset (see below) for technical reasons, such as major hardware upgrades during the study (at two sites), miscalibration of image resolution, excess movement, or failure of one or more automatic processing methods. An additional 73 individuals were removed due to missing covariate values. The main demographic and clinical data are summarized for the remaining 645 individuals in Table 1. Consent was obtained according to the Declaration of Helsinki. The Ethical Committees of each Institution, in which the work was performed, approved the study.

**Table 1:** Baseline Demographic, Clinical, and Neuroimaging Characteristics of Study Participants.

	Samples	Age	# Males (%)	Education Level	APOE ε4 (%)	GDS	CDR	MMSE	ADAS-COG
Controls	175	76.1 (4.9)	96 (54.8)	16.2 (2.7)	55 (31.4)	1.0 (1.2)	0.00 (0.0)	29.1 (0.95)	10.18 (6.7)
MCI	316	75.4 (7.2)	204 (64.5)	15.8 (2.9)	172 (54.4)	1.54 (1.4)	0.49 (0.03)	27.1 (1.8)	11.94 (5.9)
AD	154	75.4 (7.6)	82 (53.2)	14.9 (2.9)	90 (58.4)	1.6 (1.4)	0.72 (0.23)	23.5 (2.0)	13.2 (6.3)

Values shown in parentheses are standard deviation, except where noted in the column header. Abbreviations in column headings are as follows: MCI, mild cognitive impairment; AD, Alzheimer’s disease; GDS, geriatric depression scale total score; MMSE, Mini-Mental Status Exam total score; ADAS-COG, Alzheimer’s disease assessment scale-cognitive subscale, total 11. Age and education level are measured in years.

### Genotyping

Samples were genotyped using the Illumina Human610-Quad BeadChip. The Illumina Human610-Quad BeadChip consists of 550,000 polymorphic sites (SNPs), plus an additional 60,000 genetic markers including 138 mitochondrial DNA sequence polymorphic sites. The genotyping procedure for the mtDNA is described elsewhere [23]. The 138 mitochondrial SNPs are based on the AF347015.1 mtDNA reference sequence, one of 53 African sequences deposited in Genbank [24]. We first mapped the 138 mtDNA SNPs to the revised Cambridge Reference Sequence (rCRS). The 138 SNPs consist of 21 noncoding, 91 protein-coding, 4 rRNA, 11 tRNA and 1 termination sites. No imputed SNPs were used in this research.

### ADNI Phenotypic Data

We used hippocampal and whole brain volume (WBV) data from the Anders Dale Lab (UCSD) available as part of the ADNI secondary imaging data downloads, from which we also calculated rates of hippocampal atrophy and WBV atrophy using two-year data. Details on their neuroimaging processing methods are published elsewhere [25]. For normalization calculations we used total-intracranial volumes calculated automatically from FreeSurfer [26], also available from the ADNI secondary imaging data downloads. We also chose to use several imaging biomarkers that have been elucidated in the recent literature as being sensitive to disease progression and AD genetic risk [19]. Our variables were data from the recent FreeSurfer (2012) upload on the ADNI website, and included baseline and longitudinal (2-year) percent change scores derived from cortical thickness measures in the entorhinal cortex, parahippocampal cortex, and the temporal pole. In addition, we computed a hippocampal occupancy (HOC) score as another estimate of medial temporal lobe atrophy, which may aid in differentiation of individuals with congenitally small hippocampi from those with small hippocampi due to a degenerative disorder [27]. It has been shown to have higher discriminative and predictive accuracy than the standard hippocampal volume measure [27]. The HOC was computed as the ratio of hippocampal volume to the sum of the hippocampal and interior lateral ventricular volumes in each hemisphere separately. Right and left HOC scores were then averaged. As a marker for disease-related cognitive change, we used the ADAS-Cog total 11 score at baseline and 2-year change scores for longitudinal data.

### Haplotype Network Estimation

We used TCS [28] to estimate a haplotype network using the 138 genotyped mtDNA SNPs from all 821 individuals. As part of network construction, TCS collapsed all the individuals into common haplotypes. From 821 individuals there were 196 different haplotypes. The initial network contained ambiguities (or multiple paths through the network to arrive at a given node), and we were able to resolve (or select a single path to each node) all but one of these ambiguities using the following criteria: first, observed haplotypes are more likely to be descended from internal (or more ancient) haplotypes than external (or more recent) haplotypes (in this case we select the path connecting a node to the most ancient haplotype possible), and second, haplotypes are more likely to descend from higher frequency haplotypes (in this case we select the path connecting a node to the haplotype with highest frequency possible). After resolving ambiguities using the criteria above there was still one ambiguity that was factored into our analyses. The complete network can be seen in Figure S1.

### Association Analyses

Genotype-phenotype associations were evaluated using an evolution-based method known as TreeScanning [29], [30] that makes use of haplotype networks. Haplotype networks provide a framework from which to select evolutionarily related haplotypes to pool together for comparison and makes the assumption that a variant responsible for a phenotype is embedded within the structure represented by the network. Additional details about the application of TreeScanning to a similar dataset can be found in Ridge et al. [31]. The null hypothesis of TreeScanning is that the phenotype does not differ in distribution across the genotypes derived from allelic classes defined by the branches of the haplotype network. Each branch of the haplotype network can be “cut” creating two different groups (one group on each side of the cut branch). For the sake of association testing, each of these groups is treated as a separate allele (so we have two alleles). We then test these two different alleles against each other for phenotypic differences (in this particular study we tested to see if there was a significant difference in the average phenotype of the two groups). A branch is considered a significant branch if, when cut, it creates a partition resulting in two groups with significant phenotypic differences. Because we have multiple tests that are correlated we obtained multiple-test corrected p-values by a permutation analog of the sequential step-down Bonferroni [32] with 10,000 permutations. If significant branches are found in the first round of TreeScanning, a second round of TreeScanning that tests for phenotypic differences within the groups is performed. This second round can detect phenotypic heterogeneity within the allelic classes of the significant branch. This is accomplished by creating a three-allele system and using conditional permutations that hold one of the alleles constant while subdividing the other group into two alleles [29]. We included age, gender, and apolipoprotein E (APOE) ε4 status as covariates for each analysis. Only analyses with at least five individuals in each group were performed and corrected p-values of 0.05 or less were considered significant. In general in TreeScanning, when a significant branch is identified, which corresponds to one or more sequence features, the features of that branch are the only unique difference between the significant clades and adjacent clades, and while the entire haplotype is responsible for the observed phenotype, these particular features have particular affect on the phenotype. In our research, however, since we have only genotyped 138 SNPs, the branch may or may not correspond to the functional variant. Individuals in the significant clades identified in our research all share common mitochondrial sequence features that likely explain the phenotypic change, even if the specific sequence feature was not genotyped in our dataset. A detailed description of the methods is outlined in Ridge et al. [31].

### Mitochondrial Haplotype Assignment

In this paper we refer to haplotypes observed in our data based on the 138 genotyped mtDNA SNPs as haplotypes. Haplogroups, mitochondrial haplogroups, major mitochondrial haplogroups, and sub-haplogroups refer to known mitochondrial haplogroups (i.e. H, L, N, etc.) or their sub-haplogroups (i.e. H1A, K1, etc.).

The 138 genotyped mtDNA SNPs were insufficient to use available software for mitochondrial haplogroup assignment; therefore, we examined each of the significant contrasts in our dataset manually to identify which mitochondrial haplogroups our observed haplotypes (in only the significant clades) belong to. We used the following approach to identify haplogroups: 1) Individuals in significant clades share the same or very similar haplotypes; therefore, we first identified all the variants shared by the individuals in these clades; 2) Using phylotree.org [33], which is the most comprehensive mtDNA tree available and is based on the primary literature, we identified the different mitochondrial haplogroups known to be associated with each of the variants identified in step 1; 3) We looked for haplogroups known to be associated with all of the variants identified in step 1 and in some cases this allowed us to identify a single plausible haplogroup based on the genotyped variants in our dataset (for example, in order to name a haplogroup for a given clade, every variant present in that clade would have to be known to be associated with the named haplogroup). In some cases we were able to narrow potential haplogroups to a probable group, while in other cases there were multiple plausible haplogroups. In the instances where we identified a likely haplogroup we report it, otherwise we report no haplogroup assignment. It should be noted that our haplogroup assignments are based on 138 SNPs and are the best estimates possible with the available data.

### Ethics Statements

All study procedures were approved by the Institutional Review Boards of Brigham Young University and Kansas University. Detailed information about patient consenting can be found on the ADNI website at: www.adni-info.org.

## Results

We used TreeScanning to group the observed haplotypes in our dataset into evolutionarily meaningful groups (see methods), and tested them for association with 16 different imaging phenotypes (Table S1) carefully selected to minimize correlations between phenotypes. Pairwise correlation coefficients for the 16 phenotypes are reported in Table S2. We found evidence for an association between four mitochondrial clades and three of the brain imaging endophenotypes; whole brain volume, percent change in temporal pole thickness over two years, and left hippocampal atrophy over two years.

### Mitochondrial Haplotypes Associated with Whole Brain Volume

**Figure 1:**
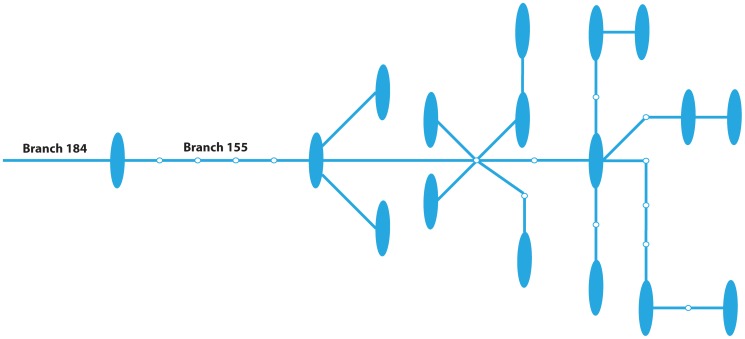
Branches 184 and 155. This is a subset of the full haplotype network (Figure S1). The large blue ovals represent observed haplotypes within our dataset and the small white nodes ancestral (and unobserved) haplotypes in the network. Each blue segment between observed or unobserved nodes corresponds to a single SNP. Only branches 184 and 155 are labeled, as these were the only branches defining groups associated with a given phenotype. Mitochondrial haplogroups were indistinguishable for the haplotypes in these clades.

The clade represented by branch 155 (Figure 1) was significantly associated (corrected p-value 0.025) with whole brain volume after correcting for age, gender, and both with and without correction for APOE ε4 status (Table 2, Figure S2, Table S3). The clade defined by branch 155 is a large clade with 16 distinct haplotypes and 18 individuals (seven controls, six mild cognitive impaired (MCI), and five AD cases). Individuals in this clade had lower whole brain volume (919170 versus 989295 in the rest of the network). Major haplotype groups were not distinguishable using the available SNP markers for the haplotypes in this clade (Table S4).

**Table 2:** Top five hits associated with whole brain volume.

Contrast	p-values without APOE		p-values with APOE		Group (standard dev)/Network (standard dev)
	nominal	corrected	nominal	corrected	
155	0.001	0.038	8.0e-4	0.025	919170 (69738)/989295 (85908)
206	0.015	0.45	0.011	0.35	931207 (66058)/988584 (86300)
184	0.024	0.59	0.018	0.49	942316 (97823)/988703 (85477)
31	0.071	0.91	0.057	0.85	968186 (80279)/989687 (86827)
40	0.071	0.91	0.057	0.85	989687 (86827)/968186 (80279)

The nominal and corrected p-values are reported for the two models either using APOE ε4 as a covariate (p-values with APOE) or not using APOE ε4 as a covariate (no APOE) for the top five contrasts with whole brain volume. We also report the number of males and females in the contrast and the levels of whole brain volume in the clade represented by the contrast and the rest of the network. Figure S2 shows the distribution of whole brain volume measures for the entire dataset.

Branch 184 is immediately adjacent to branch 155 (Figure 1) and the clade defined by branch 155 is contained within the clade defined by branch 184. Before multiple test correction branch 184 (20 total individuals, 18 overlapping with branch 155, an additional control and AD case) was associated with significantly lower levels of whole brain volume (nominal p-value 0.018, Table 2). These two clades are correlated and represent a single effect. In addition to whole brain volume, branch 184 was also associated (nominal p-value 0.0036, Table 3, Figure S3, Table S3) with lower levels of left hippocampal atrophy over two years. The clade defined by branch 184 had, on average, 2.98% left hippocampal atrophy over two years compared to 1.18% in the rest of the dataset. There were 17 haplotypes in this clade; however, we only had left hippocampal atrophy measurements for five individuals (two controls, one MCI, and two AD cases). Additional individuals could increase the significance of the association. Branch 155 had fewer than five individuals with left hippocampal atrophy measurements and therefore was not tested (see Methods) for association with this phenotype.

**Table 3:** Top five hits associated with left hippocampal atrophy.

Contrast	p-values without APOE		p-values with APOE		Group (standard dev)/Network (standard dev)
	nominal	corrected	nominal	corrected	
184	0.0026	0.064	0.0036	0.0909	−2.98 (2.00)/−1.18 (1.33)
146	0.0352	0.5562	0.0549	0.7031	−1.19 (1.33)/−2.27 (2.26)
75	0.055	0.699	0.0637	0.771	−0.281 (1.49)/−1.23 (1.35)
67	0.0822	0.8479	0.0846	0.8295	−0.76988 (1.42)/−1.252 (1.34)
23	0.1069	0.9134	0.1332	0.9483	−1.87 (1.92)/−1.19 (1.33)

The nominal and corrected p-values are reported for the endophenotype as described in Table 2. Values in the Group/Network column correspond to left hippocampal atrophy. Figure S3 shows the distribution of left hippocampal atrophy measures for the whole dataset.

### Mitochondrial Haplotypes Associated with Percent Change in Temporal Pole Thickness Over Two Years

**Figure 2:**
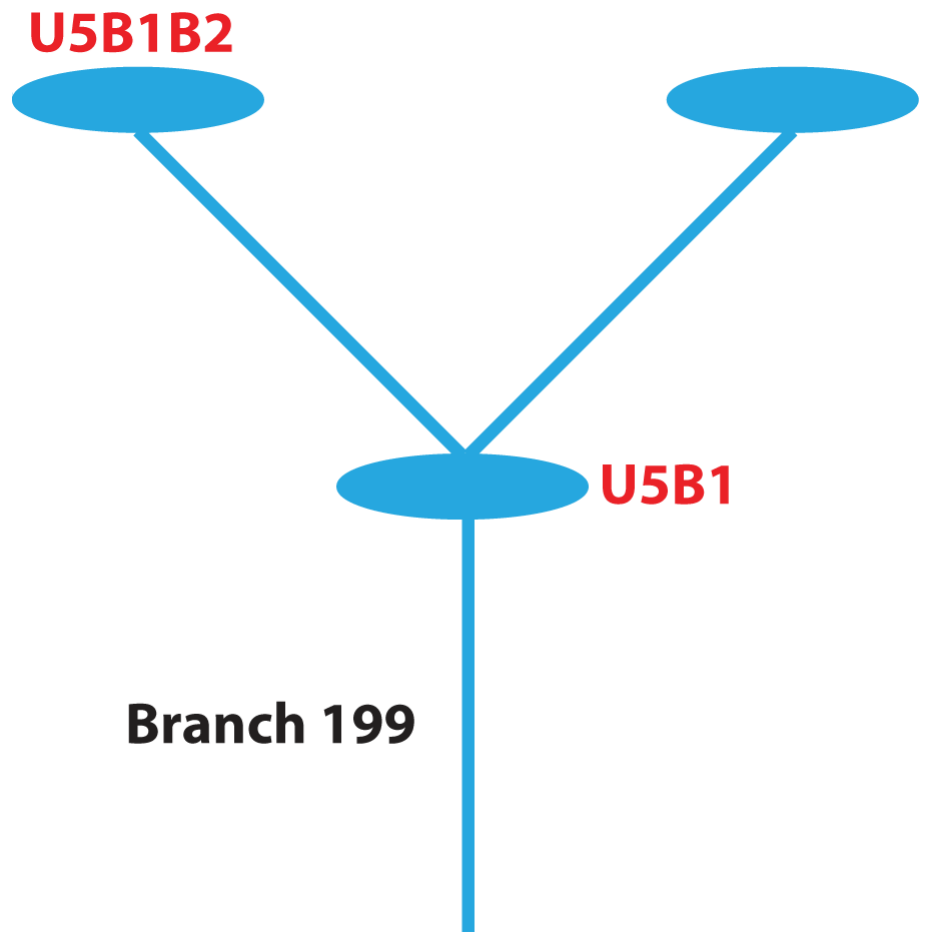
Branch 199. The nodes and colors are as described in Figure 1. Mitochondrial haplogroups were distinguishable for two of the three nodes in the clade and so are labeled.

Two different clades (Table 4, Figure S4, Table S3) were associated with higher percent change in temporal pole thickness over two years. First, the individuals in the clade defined by branch 199 (Figure 2) had an average 13.22% loss in temporal pole thickness over two years compared to 4.27% loss in the rest of the dataset (corrected p-value 0.0343). This clade consisted of three different haplotypes and five individuals (four MCI and one AD case). Based on the available SNPs we were able to identify the mitochondrial haplogroups (U5B1 and U5B1B2) for two of the three haplotypes in this clade. Branch 199 corresponds to m.5656A>G. This SNP has been reported to be specific to U5B [34], [35] or U5B1 and several other sub-haplogroups [33]. Individuals belonging to the second haplogroup, U5B1B2, also have m.217T>C. A d-loop variant, m.217T>C has been reported as being specific to U5B [35] or specific to U5B1B2 [33]. Additionally, moving one branch up the network is m.3197T>C, a variant specific to U5 [34]. While sources exist that suggest a wider distribution for these two variants, an analysis of each variant corresponding to each branch of the network as the network is descended to branch 199 clearly shows a trend towards sub-haplogroups of U (for complete details see Table S5).

**Table 4:** Contrasts associated with percent change in temporal pole thickness.

Contrast	p-values without APOE		p-values with APOE		Group (standard dev)/Network (standard dev)
	nominal	corrected	nominal	corrected	
199	0.0018	0.0416	0.0016	0.0343	−13.22 (13.12)/−4.27 (5.30)
9	0.002	0.0462	0.001	0.0441	−10.04 (7.31)/−4.10 (5.41)

The two contrasts associated with percent change in temporal pole thickness over two years are reported for the two models described in Table 2. Values in the Group/Network column correspond to percent change levels. Figure S4 shows the distribution of changes in temporal pole thickness measures for the whole dataset.

**Figure 3:**
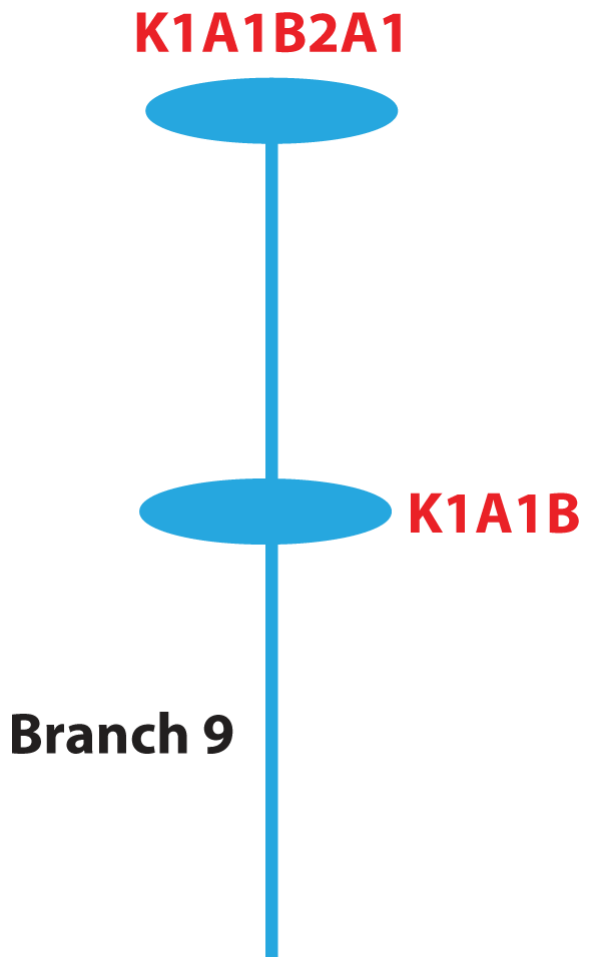
Branch 9. The nodes and colors are as described in Figure 1. Mitochondrial haplogroups were distinguishable for both nodes in the clade and are labeled.

The second clade associated with higher percent loss of temporal pole thickness over two years (corrected p-value 0.0441) is defined by branch 9 (Figure 3). The 10 individuals (three controls, four MCI, and three AD cases) in this clade had an average loss of temporal pole thickness over two years of 10% in contrast to individuals in the rest of the dataset who had an average loss in temporal pole thickness over two years of 4.079%. This clade consists of two different haplotypes corresponding to major haplogroups K1A1B and K1A1B2A1. Assignment to these haplogroups was based on several SNPs. m.10550A>G is specific to K [33], [34], m.1189T>C is one of the defining variants of K1 [34], and m.15924A>G appears in several haplogroups, one of which is K1A1B. All individuals in this clade had these three SNPs. K1A1B2A1 individuals also have m.15758A>G, a variant only observed in K1A1B2A1 [33]. After examining the possible haplogroups for the individuals in this clade, these K sub-haplogroups are the only ones that make sense given the available SNP data (Table S6).

In addition to percent change in temporal pole thickness, these two branches were also nominally associated with several other phenotypes. Branch 199 was nominally associated with lower levels of hippocampal occupancy (at their 2 year scan) (p-value 0.0027), baseline hippocampal occupancy score (p-value 0.0055), percent change in entorhinal cortex thickness over two years (p-value 0.0073), bilateral entorhinal cortex thickness (baseline) (p-value 0.0078), temporal pole thickness (baseline) (p-value 0.0216), and percent change in HOC over two years (p-value 0.0339). Branch 9 was nominally associated with percent change in HOC over two years (p-value 0.0164), and percent change in entorhinal cortex thickness over two years (p-value 0.02).

## Discussion

Here we present evidence of association between evolutionarily related groups of haplotypes and markers of AD-related neurodegeneration. We identified groups associated with whole brain volume, temporal pole thickness, and left hippocampal atrophy over two years. Association studies of mitochondrial genes and AD risk have identified a variety of mtDNA haplogroups or polymorphisms that may influence AD risk, with sometimes conflicting results. Previously reported associations are listed in Table 5. Haplogroups are defined by ancestral polymorphisms that are continent-specific, and nine primary mitochondrial haplogroups have been identified in European populations [36]. Two of these haplogroups, specifically H and U, and also the UK cluster (a study performed on a subset of the dataset used for the present research), have been most often implicated in risk for AD [37]–[40] although a sub-clade of H has also been associated with protection from AD [31]. Our data confirm and extend studies, including one based on some of these same data, showing that clades in haplogroups U and K and the UK cluster may be involved in AD risk. We found that individuals from two clades belonging to either U5B1 or U5B1B2 (in the first clade, branch 199), or K1A1B or K1A1B2A1 (in the second clade, branch 9) had grater rates of temporal pole atrophy, an endophenotype of AD risk [41], [42]. Each of these clades was also associated with several other endophenotypes, and point to specific mitochondrial haplogroups controlling multiple facets of brain physiology.

**Table 5:** Mitochondrial haplogroups associated with Alzheimer’s disease.

Haplogroup/Cluster	Risk Haplogroup	Protective Haplogroup	Strength of Association(s)	Ethnic Group(s)
B4C1	[60] *		Not reported	Japanese
G2A	[60] *		Not reported	Japanese
HV	[61]		0.032	Polish
H	[37], [62]		0.016/0.001	Iranian/Spanish
H5/H5A	[38], [39] *		0.0327/Not reported	Italian/European
H6A1A/H6A1B		[31]	0.017	Caucasian
K		[63] **	Not reported	Italian
N9B1	[60] *		Not reported	Japanese
U	[37], [64]	[63], [64] **	0.0003/0.04/Not reported/0.007	Iranian/Caucasian/Italian/Caucasian
UK	[40]		0.013	Caucasian
No Reported Association [65]–[70]				

Here we list previously reported associations (or lack thereof) of mtDNA with Alzheimer’s disease.

* P-values not reported for the association.

** Specific p-values were not reported, but the haplogroups (K and U) neutralized the harmful effect of the ε4 allele.

We used several brain imaging endophenotypes commonly used in Alzheimer’s disease to characterize disease progression, and found that temporal pole atrophy and left hippocampal atrophy were most significantly associated with mitochondrial genetic variation that has been implicated in risk for AD. The hippocampal formation is a key structure involved in learning and memory [43], and is highly heritable in humans [44], [45]. It has thus become an important phenotype for the measurement of Alzheimer’s disease risk and progression, specifically, measurements of change in volume and density over time, as it is one of the first structures to degenerate [46]. Change in hippocampal volume is not only associated with risk for AD, but may also be mediated by risk genes for Alzheimer’s disease such as APOE (reviewed by [47]), as well as affected by risk factors such as a maternal family history of AD [48]. In our study, the impact of mitochondrial genetic variation on hippocampal atrophy is independent of APOE ε4 status. The temporal pole, or rostralmost portion of the temporal lobe, is also known to be a location of pathology of AD [49]. Lower temporal lobe volume has been identified as a biological marker and risk factor for MCI and AD [50]. Temporal lobe volume has also been strongly associated with a single-nucleotide polymorphism in the gene GRIN2B, which has been implicated in learning and memory as well as characteristic features of AD neurodegeneration [42]. Moreover, temporal pole thickness (at baseline scan) was significantly related to genetic variation in several AD risk genes, including APOE, CNTN5 (rs10501927), and BIN1 (rs7161528) in a recent study of ADNI data [19]. Thus, our data adds to the growing literature characterizing genetic involvement in AD-related brain change by pointing towards additional mitochondrial genetic variation that may be contributing to neurodegeneration.

In addition to characterizing the impact of risk genes on brain imaging phenotypes of AD [51], we recently found that individuals with a maternal family history of AD have reductions in gray matter volume in AD-vulnerable brain regions at baseline, and that these same healthy individuals have progressive gray matter volume reductions in select AD-vulnerable brain regions over two years, independent of APOE ε4 status [5], [52]. In addition, we recently reported that individuals within the ADNI dataset with a maternal history of dementia had increased AD biomarkers, including increased global PiB uptake, lower cerebrospinal fluid (CSF) amyloid-β and a higher tau/amyloid-β ratio than individuals with a paternal, or no family history of AD [7]. These data complement other studies of maternal inheritance of AD, mtDNA mutations, and cytochrome oxidase deficits in AD [1]–[6], [53]. The possibility mtDNA might differ between AD case and control subjects is further supported by studies of cytoplasmic hybrids (cybrids) [54], [55]. Furthermore, AD-related increases in mtDNA mutations are found in brain regions first affected by the disease process [56]. While genetic variation in mtDNA is not fully responsible for transmission of AD, our current data adds to a growing literature pointing towards involvement of mtDNA in AD-related neurodegeneration and AD-risk. The mitochondrial cascade hypothesis of sporadic AD proposes that a person’s genes, both nuclear and mitochondrial, arbitrate their baseline mitochondrial function and sustainability. In this model, mitochondrial function declines with age, and at a certain level of cellular dysfunction, amyloid-β production and other histological changes typical of AD arise as a consequence of compensation, and failed compensation, leading to neurodegeneration [57]–[59]. We did not investigate the relationship between mitochondrial genotype and amyloid-β, however, the association between inherited mitochondrial DNA signatures and AD endophenotypes suggests that mitochondrial function may influence AD risk via mechanisms residing upstream of beta-amyloid deposition or production. This association, therefore would be more consistent with a mitochondrial cascade hypothesis as opposed to an amyloid cascade hypothesis.

We did not include family history status as a factor in this Treescanning haplogroup approach because family history data was incomplete in many individuals, thus limiting power in this already complex analysis. However, a question for future research should be whether individuals with a maternal family history of AD are more often in clades U or K, risk clades associated with AD, and if those individuals harbor longitudinal changes in brain structure that we found associated with those mitochondrial haplogroup clades.

Our analyses were by limited by the relatively small number of genotyped SNPs. Based on available genotype data we were able to identify likely mitochondrial haplogroups for the majority of the haplotypes in significant clades; however, without complete sequence data we cannot unambiguously assign haplotypes to major haplogroups or, in some cases, even assign probable haplogroups. Besides not being able to assign individuals to mitochondrial haplogroups, without complete mtDNA sequencing it is impossible to identify likely functional variants, whereas with full mtDNA sequencing and analysis with TreeScanning, which “cuts” one branch of the haplotype network at a time, we would know which variants are most likely to explain the observed phenotypic differences. Another limitation is that the clades associated with significant changes in imaging phenotypes were comprised of limited numbers of subjects. Although we used corrected p-values and stringent permutation testing, and our data appear to complement a pattern already in the literature, it will be important to replicate these findings in a larger sample with imaging and mitochondrial and genetics data. Finally, it is also important to note that the imaging phenotypes we studied may not only point to risk for AD, but also denote accelerated aging effects that may be more general in nature and not specific to AD.

In conclusion, using 138 genotyped SNPs in the ADNI dataset, we estimated a haplotype network and used an evolution-based approach, TreeScanning, to look for haplotypes or clades associated with 16 different imaging phenotypes. We found clades associated with significant changes in three phenotypes: whole brain volume, temporal pole thickness, and left hippocampal atrophy over two years. In addition to these three phenotypes these same clades were nominally associated with additional phenotypes, providing suggestive evidence of clades with mtDNA variants driving multiple physiological changes in several different regions of the brain related to AD. AD is a heterogenous disease and these data provide additional evidence of a role for mtDNA in the risk of AD. Future studies should focus on denser SNP genotyping, preferably whole mtDNA sequencing, and be performed in families showing evidence for a maternal transmission of AD.

## Supporting Information

Figure S1 **Haplotype network.** This is the haplotype network constructed using the 138 genotyped SNPs. The four branches, which define the clades associated with phenotypes in this study, are labeled.
(TIF)

Figure S2 **Distribution of whole brain volume measurements.** We note that our p-values were calculated using permutation (a non-parametric approach). Therefore, the distribution does not affect the validity of the tests.
(TIFF)

Figure S3 **Distribution of left hippocampal atrophy measurements.** See the legend to Figure S2.
(TIFF)

Figure S4 **Distribution of percent change in temporal pole thickness measurements.** See the legend to Figure S2.
(TIFF)

Table S1 **Tested phenotypes.** This is a list of the 16 phenotypes we tested for association with mtDNA. Change measures are for 2 year longitudinal data. Volumes were normalized. HOC; Hippocampal Occupancy Score.
(DOCX)

Table S2 **Pairwise correlation coefficients.** Here we report pairwise correlation coefficients for each of the tested phenotypes, and corresponding p-values. Cells highlighted yellow are significant correlations. While there are many significant correlations, most are very small in magnitude.
(XLSX)

Table S3 **Single SNP association tests.** Analysis of covariance (ANCOVA) was performed in SAS to identify possible variant association to the phenotypes of interest. Covariants in the analysis were: age, gender, number of APOE alleles, and the SNP of interest. A significance threshold for 138 mitochondrial variants is an alpha of 0.0003. The regression sum of squares statistic was calculated for each mitochondrial variant. No significant SNPs asociations were identified. In each of the tabs, the SNP position listed is the array SNP position. The last tab, “Position Mappings”, has a key to translate the array position to NC_012920.
(XLSX)

Table S4 **Mitochondrial haplogroups for the clade defined by branch 155.** Mitochondrial haplogroups associated with each of the listed variants. Variants are listed (top to bottom) as the haplotype network is descended from the root, so each variant, top to bottom, is specific to a smaller set of observed haplotypes in our dataset. Haplogroups listed in red experience a back mutation of the listed variant, so they have the ancestral allele as opposed to the variant allele. *These variants define branch 155.
(DOCX)

Table S5 **Mitochondrial haplogroups for the clade defined by branch 199.** Mitochondrial haplogroups associated with each of the listed variants. Variants are listed and haplogroups colored as described in Supplementary Table 2 except that highlighted (in yellow) haplogroups show the path from general (haplogroup U) to specific (haplogroups U5B1 and U5B1B2) used to assign haplogroups to the clade defined by branch 199. *This variant defines branch 199 **This variant defines one of the branches within the clade defined by branch 199.
(DOCX)

Table S6 **Mitochondrial haplogroups for the clade defined by branch 9.** Mitochondrial haplogroups associated with each of the listed variants. Variants are listed and haplogroups colored as described in Supplementary Table 3 except that we are highlighting the path to K1A1B and K1A1B2A1. *This variant shows a reverse mutation in K1; however, a review of the literature reveals there is confusion about what the ancestral allele is at this position, so the SNP chip actually found these individuals were WT (by calling the ancestral allele the variant allele) **This variant defines branch 9. ***This variant defines one of the branches within the clade defined by branch 9.
(DOCX)
