# Supplementary figures and images for: Mitochondrial Haplotypes Associated with Biomarkers for Alzheimer’s Disease
Source: PLoS One. 2013 Sep 11;8(9):e74158. doi: 10.1371/journal.pone.0074158 (PMC3770576; doi:10.1371/journal.pone.0074158)

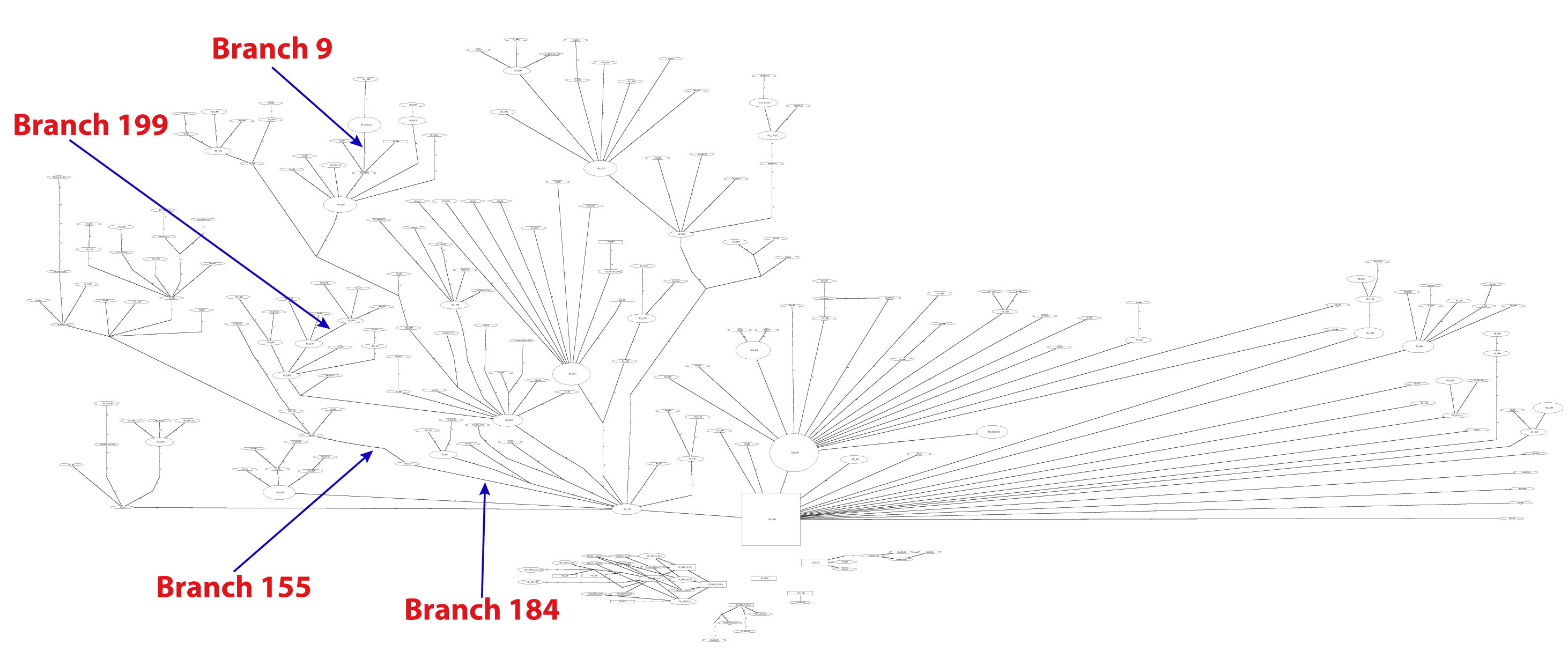

Supplement: Figure S1 — Haplotype network. This is the haplotype network constructed using the 138 genotyped SNPs. The four branches, which define the clades associated with phenotypes in this study, are labeled. (TIF) [file pone.0074158.s001.tif]

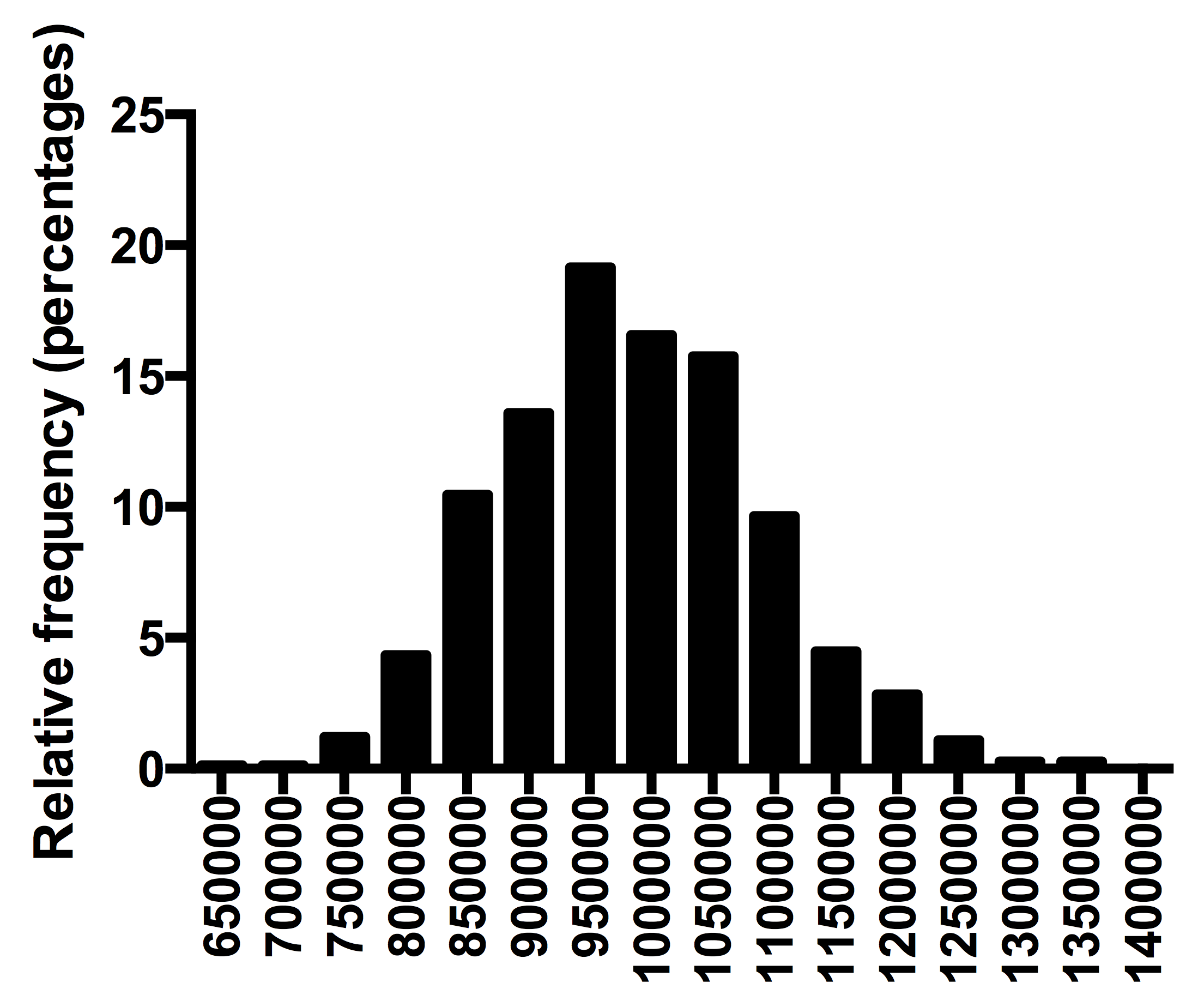

Supplement: Figure S2 — Distribution of whole brain volume measurements. We note that our p-values were calculated using permutation (a non-parametric approach). Therefore, the distribution does not affect the validity of the tests. (TIFF) [file pone.0074158.s002.tiff]

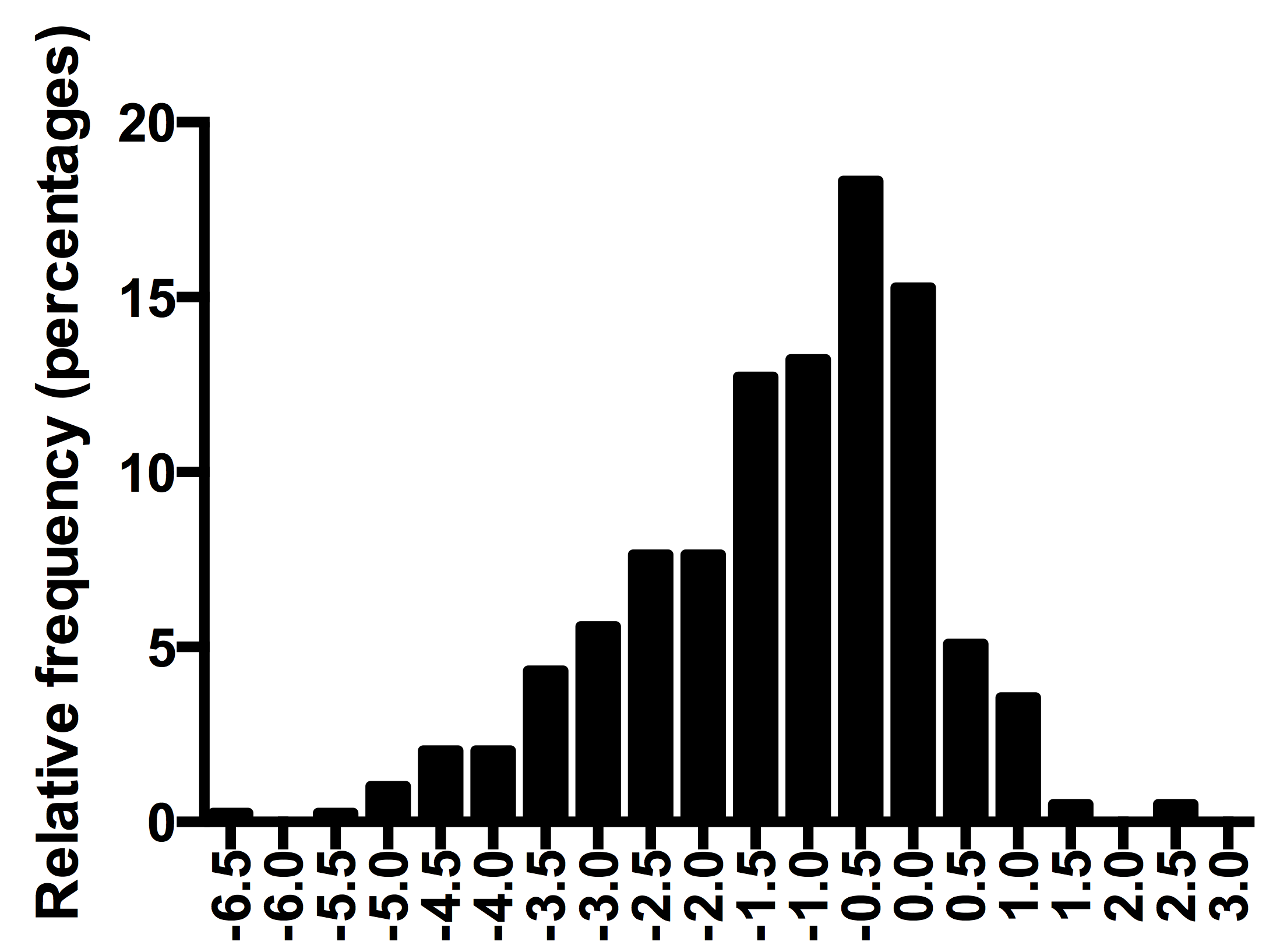

Supplement: Figure S3 — Distribution of left hippocampal atrophy measurements. See the legend to Figure S2. (TIFF) [file pone.0074158.s003.tiff]

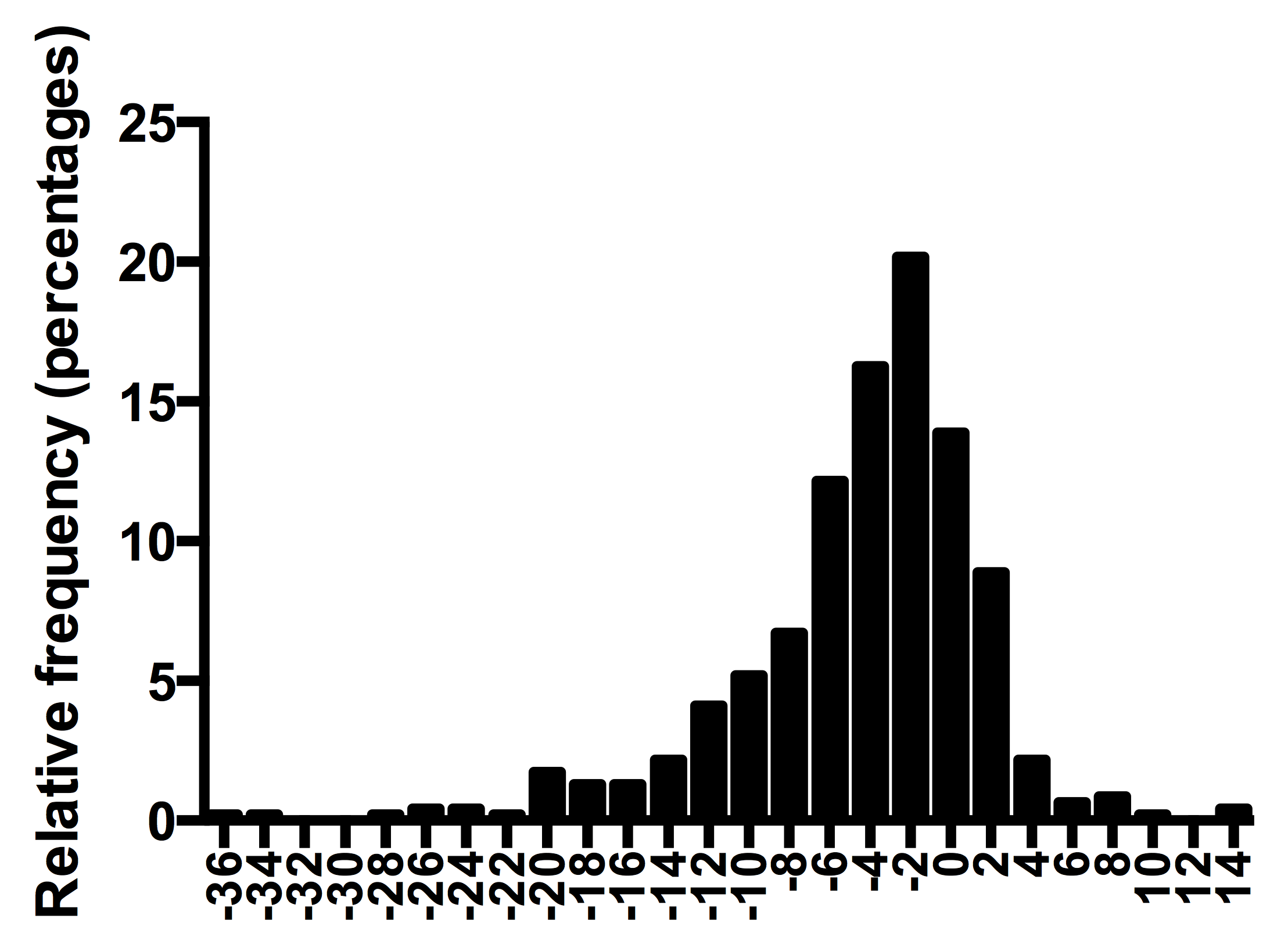

Supplement: Figure S4 — Distribution of percent change in temporal pole thickness measurements. See the legend to Figure S2. (TIFF) [file pone.0074158.s004.tiff]
