# Supplementary material for: Mitochondrial Haplotypes Associated with Biomarkers for Alzheimer’s Disease
Source: PLoS One. 2013 Sep 11;8(9):e74158. doi: 10.1371/journal.pone.0074158 (PMC3770576; doi:10.1371/journal.pone.0074158)
Supplement: Table S1 — Tested phenotypes. This is a list of the 16 phenotypes we tested for association with mtDNA. Change measures are for 2 year longitudinal data. Volumes were normalized. HOC; Hippocampal Occupancy Score. (DOCX) [file pone.0074158.s005.docx]

**Table S1.** Tested phenotypes.

| **Phenotypes** |
| --- |
| ADAS Cog Total 11 Score Baseline |
| % Change in ADAS Cog Total 11 Score |
| Whole Brain Volume (Baseline) |
| Left Hippocampal Volume (Baseline) |
| Right Hippocampal Volume (Baseline) |
| Annualized Whole Brain Volume Atrophy |
| Annualized Left Hippocampal Volume Atrophy |
| Annualized Right Hippocampal Volume Atrophy |
| Average HOC (Baseline) |
| % Change HOC |
| Parahippocampal Cortex Thickness (Baseline) |
| % Change Parahippocampal Cortex Thickness |
| Entorhinal Cortex Thickness (Baseline) |
| % Change Entorhinal cortex |
| Temporal Pole Thickness (Baseline) |
| % Change Temporal Pole Thickness |

This is a list of the 16 phenotypes we tested for association with mtDNA. Change measures are for 2 year longitudinal data. Volumes were normalized. HOC; Hippocampal Occupancy Score
