# Supplementary material for: Mitochondrial Haplotypes Associated with Biomarkers for Alzheimer’s Disease
Source: PLoS One. 2013 Sep 11;8(9):e74158. doi: 10.1371/journal.pone.0074158 (PMC3770576; doi:10.1371/journal.pone.0074158)
Supplement: Table S4 — Mitochondrial haplogroups for the clade defined by branch 155. Mitochondrial haplogroups associated with each of the listed variants. Variants are listed (top to bottom) as the haplotype network is descended from the root, so each variant, top to bottom, is specific to a smaller set of observed haplotypes in our dataset. Haplogroups listed in red experience a back mutation of the listed variant, so they have the ancestral allele as opposed to the variant allele. *These variants define branch 155. (DOCX) [file pone.0074158.s008.docx]

**Table S4.** Mitochondrial haplogroups for the clade defined by branch 155.

| **Variant** | **Possible Haplogroups** |
| --- | --- |
| m.2706A>G | L0D1, M23, M45A, D4F1, A4F, J1C3C, H, U2B |
| m.9950T>C | L0D1A , L3F1, M11, W1F, B5, B2 |
| m.15301G>A* | L1C1D, L2, L3, L4, L6 |
| m.12705C>T* | R |
| m.10873T>C* | N |
| m.10398A>G* | L1C1A, L3E1A3, N, J1C8, N8, Y, N1A, N1E, N1EI, I, R11, B4C1C, B5, R12, R21, P4, J, R0A2K1, K1 |
| m.9540T>C* | N |

Mitochondrial haplogroups associated with each of the listed variants. Variants are listed (top to bottom) as the haplotype network is descended from the root, so each variant, top to bottom, is specific to a smaller set of observed haplotypes in our dataset. Haplogroups listed in red experience a back mutation of the listed variant, so they have the ancestral allele as opposed to the variant allele.

*These variants define branch 155
