# Supplementary material for: Mitochondrial Haplotypes Associated with Biomarkers for Alzheimer’s Disease
Source: PLoS One. 2013 Sep 11;8(9):e74158. doi: 10.1371/journal.pone.0074158 (PMC3770576; doi:10.1371/journal.pone.0074158)
Supplement: Table S5 — Mitochondrial haplogroups for the clade defined by branch 199. Mitochondrial haplogroups associated with each of the listed variants. Variants are listed and haplogroups colored as described in Supplementary Table 2 except that highlighted (in yellow) haplogroups show the path from general (haplogroup U) to specific (haplogroups U5B1 and U5B1B2) used to assign haplogroups to the clade defined by branch 199. *This variant defines branch 199 **This variant defines one of the branches within the clade defined by branch 199. (DOCX) [file pone.0074158.s009.docx]

**Table S5.** Mitochondrial haplogroups for the clade defined by branch 199.

| **Variant** | **Possible Haplogroups** |
| --- | --- |
| m.2706A>G | L0D1, M23, M45A, D4F1, A4F, J1C3C, H, U2B |
| m.12372G>A | M7C1D, M12, D4H1A1, N9A, U, H4AU |
| m.12308A>G | U, U5A2B2 |
| m.11467A>G | U |
| m.3197T>C | L3F1A1, H14B, U2E1A1A, U5 |
| m.16270C>T | L1B, L3H1A2B, M58, M2A1, M13B2, M61, N11B, P1D1, H1BA, U5B2A1, K2B1A, U5 |
| m.7768A>G | U5B |
| m.5656A>G* | L5A, M26, T2B3A, HV1B1B, U5B1 |
| m.217T>C** | L3B1B1, H3H4, U5B1B2, U2E1, U2E2, U2E3, K1A4A1F1 |

Mitochondrial haplogroups associated with each of the listed variants. Variants are listed and haplogroups colored as described in Supplementary Table 2 except that highlighted (in yellow) haplogroups show the path from general (haplogroup U) to specific (haplogroups U5B1 and U5B1B2) used to assign haplogroups to the clade defined by branch 199.

*This variant defines branch 199

**This variant defines one of the branches within the clade defined by branch 199
