# Supplementary material for: Mitochondrial Haplotypes Associated with Biomarkers for Alzheimer’s Disease
Source: PLoS One. 2013 Sep 11;8(9):e74158. doi: 10.1371/journal.pone.0074158 (PMC3770576; doi:10.1371/journal.pone.0074158)
Supplement: Table S6 — Mitochondrial haplogroups for the clade defined by branch 9. Mitochondrial haplogroups associated with each of the listed variants. Variants are listed and haplogroups colored as described in Supplementary Table 3 except that we are highlighting the path to K1A1B and K1A1B2A1. *This variant shows a reverse mutation in K1; however, a review of the literature reveals there is confusion about what the ancestral allele is at this position, so the SNP chip actually found these individuals were WT (by calling the ancestral allele the variant allele) **This variant defines branch 9. ***This variant defines one of the branches within the clade defined by branch 9. (DOCX) [file pone.0074158.s010.docx]

**Table S6.** Mitochondrial haplogroups for the clade defined by branch 9.

| **Variant** | **Possible Haplogroups** |
| --- | --- |
| m.2706A>G | L0D1, M23, M45A, D4F1, A4F, J1C3C, H, U2B |
| m.12372G>A | M7C1D, M12, D4H1A1, N9A, U, H4AU |
| m.12308A>G | U, U5A2B2 |
| m.11467A>G | U |
| m.9698T>C | H1B1E, U8 |
| m.3480A>G | B5B1C, U8B, UK |
| m.10550A>G | K |
| m.14798T>C | J1C, T2G, K |
| m.1189T>C | U4A2F, K1 |
| m.10398A>G* | L1C1A, L3E1A3, N, J1C8, N8, Y, N1A, N1E, N1EI, I, R11, B4C1C, B5, R12, R21, P4, J, J1C8, R0A2K1, K1 |
| m.11914G>A | L1, L2, L3, L4, L5, L6, M20, M5A5, C, G1A1A, G3A1, M77, L1C1D, L2A1-L2A4, D1A2, D1G4, D4H3A1A2, N1C, A2F3, X2B6, T2C1A, R8A1A1C, B6A, B4B1C1, B4D, B5B1B, R23, P2, H1H1, H1N4, H1AO1, H3X1, H15A1B, U8B1A1, K1A1, K1B1C, K2A2A1 |
| m.15924A>G** | U5A1A1, U5B2A1A2, K1A1B, L1C3B2, L2A1C2, L3E2A1A, M2A1B, C4B7, M13, M21A, M35A, D1G4, D4E1A, D4N1, N1E, I, N8, A2K1, A2X, O1A, X2G, B4A1A1B, B2J, P10, R0A2B, H1N1B, M13B2 |
| m.15758A>G*** | K1A1B2A1, L2A5, L3I1A, L3X2B, G2A1B, I2, B5B2B, H1C14, H1AT, H13A1A2B, H20B, H28A1 |

Mitochondrial haplogroups associated with each of the listed variants. Variants are listed and haplogroups colored as described in Supplementary Table 3 except that we are highlighting the path to K1A1B and K1A1B2A1.

*This variant shows a reverse mutation in K1; however, a review of the literature reveals there is confusion about what the ancestral allele is at this position, so the SNP chip actually found these individuals were WT (by calling the ancestral allele the variant allele)

**This variant defines branch 9

***This variant defines one of the branches within the clade defined by branch 9
